# Childhood Malaria Admission Rates to Four Hospitals in Malawi between 2000 and 2010

**DOI:** 10.1371/journal.pone.0062214

**Published:** 2013-04-26

**Authors:** Emelda A. Okiro, Lawrence N. Kazembe, Caroline W. Kabaria, Jeffrey Ligomeka, Abdisalan M. Noor, Doreen Ali, Robert W. Snow

**Affiliations:** 1 Malaria Public Health Department, Kenya Medical Research Institute-Wellcome Trust-University of Oxford Collaborative Programme, Nairobi, Kenya; 2 Centre for Tropical Medicine, Nuffield Department of Medicine, University of Oxford, Oxford, United Kingdom; 3 Department of Mathematical Sciences, Chancellor College, University of Malawi, Zomba, Malawi; 4 Department of Statistics, University of Namibia, Windhoek, Namibia; 5 Records Department, Zomba Central Hospital, Zomba, Malawi; 6 National Malaria control Programme, Ministry of Health, Lilongwe, Malawi; Menzies School of Health Research, Australia

## Abstract

**Introduction:**

The last few years have witnessed rapid scaling-up of key malaria interventions in several African countries following increases in development assistance. However, there is only limited country-specific information on the health impact of expanded coverage of these interventions.

**Methods:**

Paediatric admission data were assembled from 4 hospitals in Malawi reflecting different malaria ecologies. Trends in monthly clinical malaria admissions between January 2000 and December 2010 were analysed using time-series models controlling for covariates related to climate and service use to establish whether changes in admissions can be related to expanded coverage of interventions aimed at reducing malaria infection.

**Results:**

In 3 of 4 sites there was an increase in clinical malaria admission rates. Results from time series models indicate a significant month-to-month increase in the mean clinical malaria admission rates at two hospitals (trend P<0.05). At these hospitals clinical malaria admissions had increased from 2000 by 41% to 100%. Comparison of changes in malaria risk and ITN coverage appear to correspond to a lack of disease declines over the period. Changes in intervention coverage within hospital catchments showed minimal increases in ITN coverage from <6% across all sites in 2000 to maximum of 33% at one hospital site by 2010. Additionally, malaria transmission intensity remained unchanged between 2000–2010 across all sites.

**Discussion:**

Despite modest increases in coverage of measures to reduce infection there has been minimal changes in paediatric clinical malaria cases in four hospitals in Malawi. Studies across Africa are increasingly showing a mixed set of impact results and it is important to assemble more data from more sites to understand the wider implications of malaria funding investment. We also caution that impact surveillance should continue in areas where intervention coverage is increasing with time, for example Malawi, as decline may become evident within a period when coverage reaches optimal levels.

## Introduction

Malaria poses a major public health challenge in many part of sub-Saharan Africa and is recognized as part of the global health agenda as a significant barrier to achieving the Millennium Development Goal of improving child survival by 2015 [1]. In response, increased overseas development assistance (ODA) for malaria has led to a rapid increase in coverage of interventions aimed at reducing the malaria burden across Africa over the last five years [2], [3].

There is a growing body of evidence of the health impact associated with increasing flows of malaria ODA [4]–[23] and also modeled predictions on the impact on malaria mortality [24]–[29]. The largest between country evidence comes from observations of hospital admissions [30] and while limited in their geographic coverage they continue to represent a valuable empirical evidence-platform on changing patterns of disease since the launch of the Roll Back Malaria (RBM) Initiative in 2000.

Malawi is a high mortality burden [31], poor country [32], located in South Central Africa. Malawi has a high malaria burden and has received approximately 140 million USD in malaria specific ODA since 2000. Nevertheless a recent examination of malaria admission rates to the Queen Elizabeth Hospital in the south of Malawi was unable to show any changes in severe paediatric malaria between 2001 and 2010 [33]. Here we explore the impact of changing coverage of malaria control in relation to paediatric admission rates across a wider geographical area at hospital settings in four areas of Malawi and site specific changes in malaria intervention coverage.

## Methods

### Malawi Malaria Context

In comparison to other countries in sub-Saharan Africa, Malawi was slower to realize adequate donor support before 2008 [3] which inevitably resulted in a much slower scale up and lower levels of intervention coverage [34], [35]. In 2001 the Malawian Government adopted a policy on insecticide treated nets (ITN) to ensure 60% coverage by 2005. The second Malaria Strategic Plan (MSP) [35], launched in June 2005, sought to scale up interventions to ensure 80% coverage of ITNs in high risk groups and access to appropriate treatment by all those at risk of malaria by 2010. In the most recent MSP launched in 2011 these targets have been revised to achieve universal coverage of the four main malaria control interventions: long-lasting insecticide treated bed nets (LLIN), indoor residual spraying (IRS), intermittent presumptive treatment for pregnant women, and prompt treatment with effect artemisinin-based combination therapy for uncomplicated malaria by 2015 [35].

In 2000 less than 4% of Malawian children were protected by an ITN [36]. The dominant ITN delivery approach before 2007 was a combined full cost recovery retail sector promotion along with subsidized cost ITN distribution through antenatal clinics and through the community [37]. The first integrated free mass distribution of ITNs was in 2007. In July 2008 the Ministry of Health, using funds from the Global Fund (GF), launched a large-scale distribution campaign of free ITN to, newborns, children under the age of five years and pregnant women. Various household survey data have been collected in Malawi since 2000 aimed at defining the proportion of children below the age of five sleeping under an ITN during the night before the survey.

Initially IRS activities were limited to a single pilot district, Nkhotakota in 2007 covering 27,000 houses in the northern section and were subsequently repeated in 2008 and 2009. In 2010 the President’s Malaria Initiative (PMI) expanded IRS activities to the whole district this time including neighbouring Salima District. This has now been scaled-up covering approximately 500,000 households in five additional highly endemic districts in December 2010: Karonga, Nkhata Bay, Mangochi, Chikwawa, and Nsanje Districts [38].

Between 2000 and 2007 sulphadoxine-pyrimethamine (SP) was the only drug available in most government clinics [37] even though the efficacy of SP declined rapidly over this period. Officially, first line treatment policy was changed in November 2007 to the more efficacious artemether-lumefathrine (AL) but the new policy was not effectively implemented until early 2008. Additionally, in order to improve access to treatment, community based-case management of malaria was introduced in 2009 in hard to reach areas of Malawi with care provided by health assistants based in village health clinics. In spite of this in 2010 access to effective treatment remained low [34] with less than 30% of febrile children under five treated with AL in 2010 [34].

Thus within the context of financing, intervention delivery and coverage, between 2000 and 2006, there was a definite lack of significant progress toward achieving internationally agreed and nationally adopted coverage targets of malaria interventions. However, starting in 2007 there was an increase in external funding from the Global Fund (GF) and PMI, implementation of AL for treatment and a revised ITN policy to rapidly scale up ITN coverage including the use of mass campaigns.

### Selection of Hospital Sites

**Figure 1:**
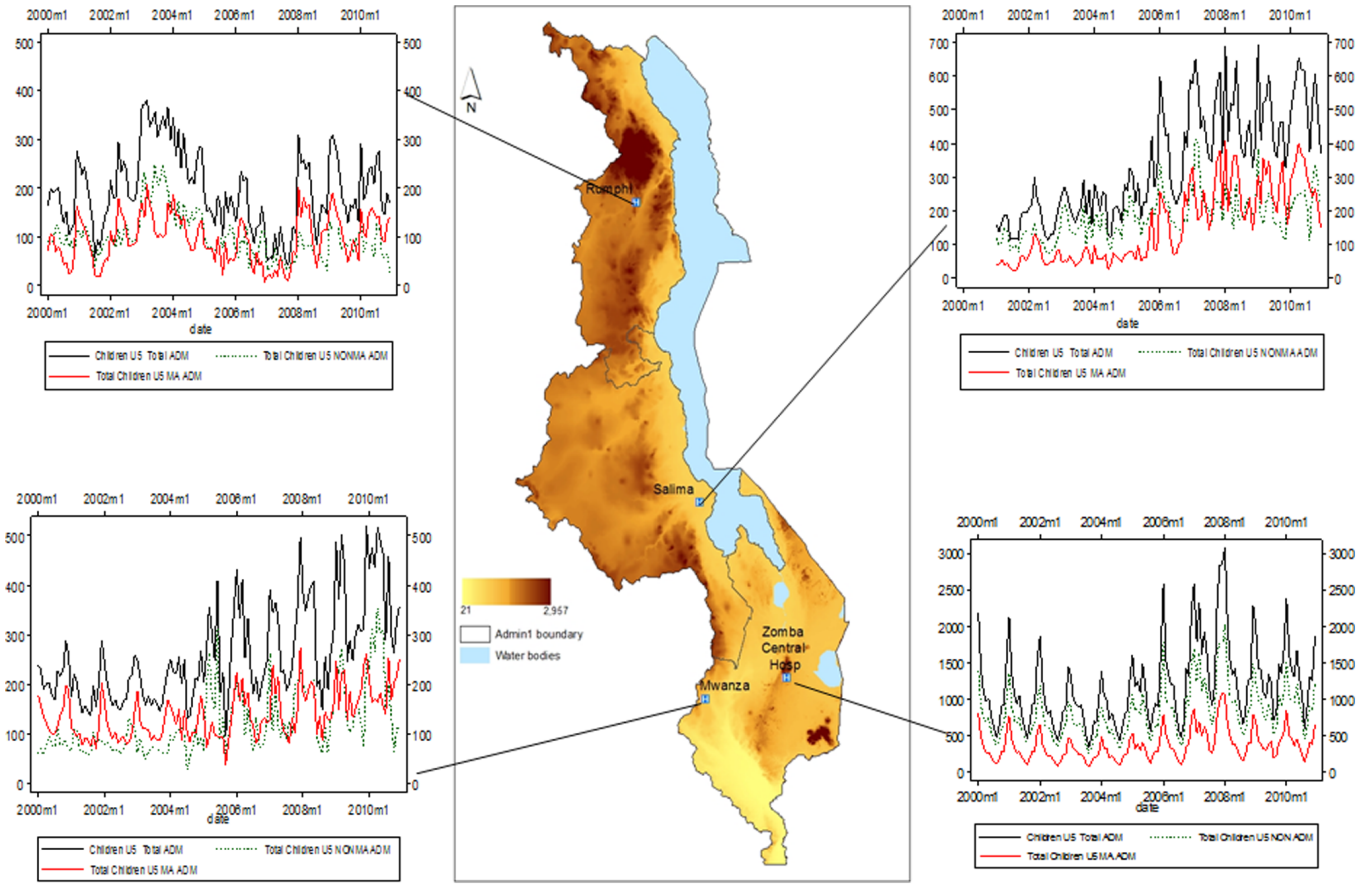
Location of selected hospitals (blue squares) shown on a 90 m digital elevation model (DEM) dataset from the Shuttle Radar Topographic Mission (SRTM) a joint project between NASA and NGA (National Geospatial-Intelligence Agency) [**77**]. Graph panels show changing paeditaric hospitalizations due to malaria (red line), non-malaria (green dotted line) and total admission in children aged 0–4 years (black line) between 2000 and 2010.

Malawi is divided into three regions: Central, Northern and Southern with a total of 28 districts. Within this there are 4 central hospitals, 22 designated district general hospitals and 46 rural hospitals managed by either the government or mission sectors. District hospitals provide secondary level health care services and serve to support peripheral clinical services by providing in-patient care and other specialized services. Despite recent efforts at improvement, traditionally routine health management information system (HMIS) reporting from all levels of service provision has been largely incomplete over long time periods and often inappropriately summarized by cause or age. To overcome the perennial inadequacies of routine HMIS data we selected a sub-set of hospitals for a more detailed inpatient review where each hospital had records available for investigation and represented the malaria ecologies typical of the country. The four hospitals selected for review are shown in Figure 1. These are located at Salima in the Central Region of Malawi along Lake Malawi and Mwanza in the South Western Region of Malawi, all areas historically supporting high, intense perennial malaria transmission; Rumphi in the Northern Region and Zomba in the Southern Region both have historically moderate risks of malaria infection [39]. The characteristics of the locations of these four hospitals are summarized in Table 1 and defined by estimates of parasite prevalence within their catchment area between 2000 and 2010 [40]. Additional information is provided on the altitude and mean average annual rainfall at each site to provide a climatic context of the risks of malaria at each site.

**Table 1:** The characteristics of malaria transmission at each hospital site.

Hospital	StartingEndemicity 1Malaria risk class	Malaria risk(*Pf*PR_2–10_) in2000, 2005,2010 2	Av. Rainfall (mm/annum2000–2006/2007–2010	Altitude^3^
Rumphi	Intermediate	35%, 35%, 36%	1268/**1149**	1205
Salima	High	41%, 41%, 42%	633/**717**	537
Mwanza	High	41%, 40%, 42%	1320/**1332**	631
Zomba	Intermediate	26%, 26%, 27%	923/**923**	937

**Notes.**

1 Endemicity classes defined as in Hay et al. [69] where >40% is accepted as highly endemic.

2 Predicted estimates of *Pf*PR from within the catchments of hospitals sites in Malawi using ARC GIS.

3 A digital elevation map (DEM) that has a resolution of 30 meters was used to define altitude (http://www.vterrain.org/Elevation/SRTM/).

### Defining Hospital Catchment Areas

We computed catchment populations to standardise the admission data across the four hospitals. Previously we have attempted to define catchment areas using addresses of admissions in Kenya [41] which were used to develop a representative catchment radius and applied to hospital data from Uganda [42]. A slightly different approach was used here. All higher level facilities (hospitals) surrounding each study site were selected from the Malawi Health facility database [43]. The spatial positions of the hospitals within a region were assembled in ArcGIS version 9.3 (ESRI, Inc., Redland, CA, USA) to define nearest distance (Thiessen) polygons around each hospital assuming that all hospitals had an equally weighted draw on patients whose distances travelled were assumed to be Euclidian (in straight lines). This assumption was necessary because the precise shape of the hospital catchments are hard to empirically define as the only measures of health service use and travel distance are available from lower order facilities providing primary out-patient care [44]–[51] and not in-patient care which is likely to have different properties [45], [48], [52]. Thiessen polygons were defined based on the bisection of the perpendicular lines of each neighbouring hospital and represent polygons whose boundaries define the area that is closest to each point (i.e. hospital) relative to all other points.

Using the lowest level of administrative units available, Traditional Authorities, all units that intersected the boundaries of the Thiessen polygon of the study hospital were included and used as the first step in defining the hospital’s catchment. In the second step, a provisional catchment was defined for each hospital using a radial distance of 30 km which had been found to capture over 90% of admissions in Kenya [41] and used to define catchments in Uganda [42]. The provisional catchment around each hospital setting were then modified depending on the mapped extents of travel barriers specifically those related to topography, bare areas, absence of road networks, and presence of non-navigable water bodies [53], [54]. Finally we used the 100×100 m population density surface [55] to calculate a convex hull polygon that enclosed the areas occupied by 90% of the catchment populations. This final area was taken to represent the study site’s physical catchment area and total population counts for the year 2000 were extracted in ARCGIS 9.3 (ESRI, Inc., Redland, CA, USA). These counts were corrected for the administrative level percentage of under-five populations and projected annually through to 2010 using sub-national inter-censal growth rates derived from the national censuses of 2008 in Malawi [56].

### Paediatric Admission Review 2000–2010

At each selected hospital, paediatric ward in-patient registers were identified for most months from January 2000 to December 2010. Each admission entry in the registers was recorded on a tally sheet indicating the month of admission and whether a primary working diagnosis of malaria had been defined for the child or whether the admission diagnosis did not include an indication of malaria. We have restricted the data assembly to records of children less than five years because they bear the burden of severe malaria in most highly endemic African countries. Individual register entries were not reconciled with patient notes and we have assumed that the admission diagnosis remained the diagnosis upon which each admission was managed clinically. Slide confirmed malaria diagnoses at admission were not universally available within, nor between hospital sites, however we have assumed that most admissions were likely to have a blood film prepared pre-admission. We remain uncertain about how, when available, parasitological results were used to guide diagnosis given the vagaries of presumed and parasitological diagnosis for in-patient paediatric case-management [57], [58]. Our working definition of “malaria” was therefore patients admitted with a diagnosis of malaria, probably managed clinically as malaria during their admission but without documented parasitological confirmation. At one site (Salima) admission data were missing for the year 2000 because registers were damaged or misplaced and therefore unavailable for review and analysis is restricted to 2001 to 2010.

This was a routine audit from registers and something undertaken as part of routine activities. The data were assembled as anonymised counts, all the data were de-identified monthly tallies of hospital admissions therefore no patient level details were included.

### Defining Hospital Catchment Endemicity and Climate

We have used parasite prevalence as a measure of transmission intensity at each hospital site selected for investigation. Parasite prevalence in children aged 2–10 years (*Pf*PR_2–10_) is a common metric of transmission intensity that is used universally and scales with paediatric malaria disease risks [59], clinical epidemiology [14], [60] and can be used to predict the impact on transmission of malaria control [61], [62]. We have used predictions of malaria transmission intensity recently developed to model the changing malaria endemicity in Malawi [40]. In brief, changes in transmission intensity between 2000 and 2010 were modeled using empirical data from over 1,000 community age-standardized parasite prevalence (*Pf*PR_2–10_) sample surveys undertaken during this period using a space-time Model Based Geostatistical framework with Bayesian inference and implemented using the Markov Chain Monte Carlo algorithm. Posterior distributions of the predicted mean *Pf*PR_2–10_ were generated at every unsampled 5×5 km grid in Malawi for the years 2000, 2005 and 2010 [40].

Rainfall is one of the most important climate variables that drive inter- and intra-annual periodicity in malaria admissions [63], [64]. To examine the effects of rainfall, an important determinant of malaria transmission, monthly precipitation data were obtained from meteorological stations located within the catchment areas of hospitals or otherwise from the nearest possible metrological station with complete data.

### Defining ITN/IRS Coverage

Data on the coverage of interventions during the study period are available from geo-located cluster sample survey data from national surveys and used here to explore the plausible correspondence between changing prevention strategies and disease incidence between hospital settings. We have used an average radial distance of 40 km around the hospitals for the purposes of obtaining a representative estimate of intervention coverage for the four Malawian hospital sites. Data on ITN and IRS coverage from clusters within a 40 km radius of each hospital were extracted in ARCGIS 9.2 (ESRI, Inc., Redland, CA, USA) from the national household sample Demographic Health Surveys (DHS) undertaken in 2000, 2004 and 2010 (Supporting Information Figure S1).

### Statistical Models

#### Time-series analysis

To examine the long-term trends in admission rates we used monthly malaria admission data, standardized for under 5 population densities within each catchment areas, with moving average smoothing methods to identify any long-term trend signals within each temporal eleven-year series while filtering out short-term annual fluctuations and random variation. The smoothing technique achieves this by replacing each element of the time series by n neighboring elements, where n is the width of the smoothing “window” equal to 12 months. We employed a centered moving average including 6 observations before and 5 after the current time point and vice versa and taking an average value of this to compute the centred average. The ARMAX model was then applied [65], which is an autoregressive model of the empirical current value of the series against one or more prior values combined with a moving average of the current value modeled against the white noise (random shocks) of one or more prior values and includes explanatory variables. The established explanatory variables for malaria trend analysis included rainfall in the current and or preceding months, assembled from meteorological stations located close to each hospital, and changes in service use (captured by non-malaria admission rates) resulting in a predicted or smoothed malaria admission rate per month for each hospital site over the period 2000 to 2010. Before specifying the ARMAX model, tests and diagnostics were performed in the estimation of the models. Monthly malaria rates were tested for stationarity using the augmented Dickey Fuller test [66] with an appropriate lag. Model diagnostics and selection criteria were used to determine the most parsimonious model. Two goodness-of-fit criteria were used to guide model selection: Akaike Information Criterion (AIC) and Schwartz’s Bayesian information criterion (BIC). The models with the lowest AIC and BIC were finally selected. All analysis was undertaken using STATA version 11.0 (Statacorp 2003, College Station, USA).

## Results

### Admission Rates

We were able to review information from registers over 132 months at 4 hospitals on 259,635 paediatric admissions for 516/528 (97.7%) admission months where 98,773 (38%) had a primary working admission diagnosis of malaria. The total admissions, clinically diagnosed malaria admissions and proportion of admissions that are clinical malaria cases are shown in Table 2 with temporal trends shown in Figure 1 (additional data presented in Table S1 Supporting information). The proportion of admission that was malaria ranged from 26% in Salima to as high as 64% in Rumphi and Mwanza between 2000 and 2010.

**Table 2:** Annual numbers of all-cause paediatric (0–4 years) admissions, malaria paediatric admissions and proportions of clinical malaria admissions for children aged less than five years by hospital site.

	2000	2001	2002	2003	2004	2005	2006	2007	2008	2009	2010
**All-Cause Admissions**											
Rumphi DGH	2019	1900	2591	4116	3209	1903	1842	956	2328	2491	2559
Salima DGH	-	1884	2229	2625	2552	3333	4604	5896	5614	5626	6408
Mwanza DGH	2624	2196	2288	2197	2532	3095	3234	3657	3481	4335	4822
Zomba CH	12652	12580	11119	9652	10177	13484	15527	23239	17720	15627	16712
Clinical Malaria **Admissions (Proportion of in-patients diagnosed at admission with clinically diagnosed malaria)**											
Rumphi DGH	873(43%)	843(44%)	1377(53%)	1669(41%)	1446(45%)	792(42%)	878(48%)	40042%)	1484(64%)	1316(53%)	1558(61%)
Salima DGH	-	520(28%)	905(41%)	687(26%)	728(29%)	1053(32%)	2118(46%)	2912(49%)	3067(55%)	3219(57%)	3537(55%)
Mwanza DGH	1686(64%)	1227(56%)	1264(55%)	1403(64%)	1496(59%)	1244(40%)	1744(54%)	1835(50%)	1822(52%)	2294(53%)	2279(47%)
Zomba CH	4025(32%0	3975(32%)	3336(30%)	2860(30%)	3147(31%)	3994(30%)	4616(30%)	7633(33%)	5511(31%)	4861(31%)	5139(31%)

Data for Salima hospital in 2000 was not available.

### Time Series Analysis

**Figure 2:**
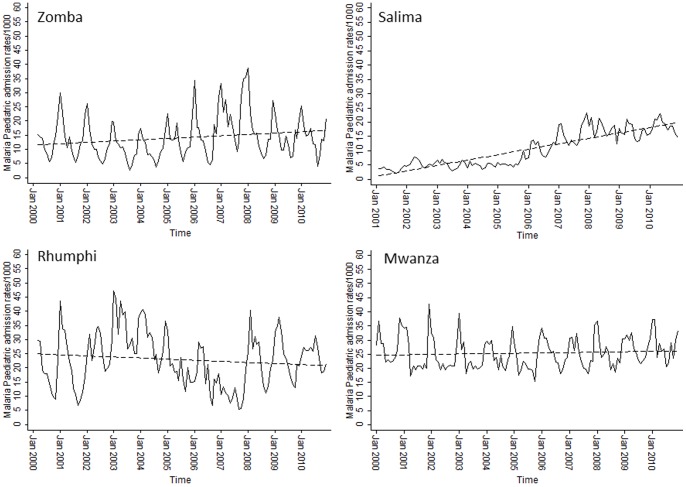
Model predictions of paediatric malaria hospitalization rates in children under 5 controlling for non-malaria case rates, rainfall and controlling for autoregressive and moving average effects (solid black line). Fitted lines illustrate the linear trends from model predictions (dashed line).

To examine the long-term temporal signals within the monthly time-series several model forms were explored. The predictions from the best fitting model that adjusted for non-malaria admission rates and rainfall at different lags while controlling for auto-regressive and moving average effects within the data are shown in Figure 2. We included linear trend lines fitted to estimated adjusted average monthly changes in clinical malaria admissions. The relationship between clinical malaria admissions by month show increases across three of the four sites; at Mwanza malaria admission rates appear to be stable across the time period. Significant (P<0.05) upward trends in malaria admission rates were observed in Salima and Zomba over the study period (Figure 2). At Rumphi rates declined between 2005 and 2007 and increased in 2008.

### Changes in Parasite Prevalence

We have summarized parasite prevalence estimates across three time points in the 11 year period using average predicted estimates of *Pf*PR_2–10_ from within the defined catchment areas of each hospital (Table 1). At the beginning of the observation period, before scaled intervention coverage in 2000, the *Pf*PR_2–10_ was highest in the Central and Southern region around Salima and Mwanza districts. Around Salima and Mwanza District General Hospitals (DGH) mean *Pf*PR_2–10_ was 41%, however it was predicted to be lower in Zomba during this period (mean *Pf*PR_2–10_, 26%; Table 1). Nearer the Northern highlands at Rumphi mean *Pf*PR_2–10_ was of intermediate level at 35%. Modelled predictions of parasite prevalence did not change at all and by the end of the period (2010) mean *Pf*PR_2–10_ had increased marginally or remained unchanged in all the four sites (Table 1).

### Changes in Intervention Coverage

The scaling-up of ITN delivery was only witnessed after 2006 in Malawi as evidenced by net distribution data [Table 2]. In Malawi free ITN mass distribution campaigns were undertaken in 2008/9. Before this, net distribution figures and coverage were largely through supplying ITN through the community and routine ANC visits or private sector distribution initially at a subsidized charge. Net deliveries per person at stable malaria risk were 0.10 in 2007 reaching 0.33 in 2010. The DHS survey undertaken in 2000 showed that ITN coverage (proportion of all household members reporting to have slept under an ITN the night before the survey) was very low and was reported to be between 2.1% and 5.9% in the regions surrounding the four hospitals. By the end of 2005 coverage had only increased moderately to between 19% and 21% with the exception of Mwanza where coverage remained below 10%. By 2010 the proportion of the entire population sleeping under an ITN the night before the survey had increased to just over 30% in three of the four locations: Mwanza, Zomba and Salima (Table 3).

**Table 3:** ITN coverage among individual of all age groups and IRS coverage in the last 12 months.

	ITN Coverage (All Ages)^1^[n/N (examined)]		
	DHS 2000 [36]	DHS 2004 [76]	DHS 2010 [67]
Rhumphi	2.6%[8/302]	19.0%[168/885]	24.3%[944/3,892]
Salima	5.2%[87/1,666]	21.1%[907/4,290]	32.8%[1,529/4,662]
Mwanza	2.1%[9/435]	9.0%[75/833]	31.9%[2,313/7,251]
Zomba	5.9%[177/3,015]	19.6%[1,150/5,856]	30.3%[2,901/9,587]

**Notes:**

Data on ITN and IRS coverage from clusters within a 40 km radius of each hospital were extracted in ARCGIS 9.2 (ESRI, Inc., Redland, CA, USA) from the Malawian DHS dates of 2000, 2004 and 2010.

1 ITN Coverage- Coverage defined as the proportion of individuals of all ages who slept under an ITN the night before the surveys.

ITN- Insecticide treated net; DHS - Demographic Health surveys, HH-Households.

In Malawi IRS activities expanded in several districts at the end of 2010 but only included one of the study districts in 2010, Salima. There are little data on the household spraying coverage but household survey data from the DHS in December 2010 suggests that 1.1% (11/971) households in the vicinity of Salima District Hospital had been sprayed within the preceding 12 months.

The proportions of reported paediatric fevers in the last 14 days recorded during the most recent household surveys that were treated with an ACT remains low. Between 2007 and 2012, there have been two surveys that sought to evaluate malaria case management practices for febrile children below 5 years of age [34], [67]. Data from the national malaria indicator survey suggests that the majority of febrile children did not access an ACT in 2010 with a reported coverage of only 28% compared to a reported 36% who were treated with an ACT from the DHS in 2010.

## Discussion

Since the launch of RBM in 2000 there has been a slow increase in external donor support and its translation into effective disease control and prevention coverage in Malawi, until important changes in access to malaria ODA and support for drug policy change and ITN distribution after 2007. Paediatric clinical malaria admissions at four hospitals across the country were analysed over 11 years, 2000 to 2010, to explore whether annualized, catchment population standardized admission rates had declined following the launch of the first NMS in 2001 and following significant changes in intervention coverage.

At three of the four hospital sites investigated, malaria admission rates have increased or remained unchanged since the period of pre-scaled intervention and funding (2000–2006) through to a period when Malawi had received improved financial support and made progress towards malaria control (2007–2010). Conversely, with the exception of Salima, non-malaria pediatric admissions remained relatively constant. Analysis of monthly time-series malaria admission rates using autoregressive models adjusting for external factors shows a significant increase in paediatric admissions from January 2000 through to December 2010 (Figures 2; linear fits P<0.05) at two of the four sites: in Salima and Zomba.

In general, malaria paediatric admission rates do not appear to be on a decline at any of the four sites investigated. At each site average starting *P. falciparum* transmission intensity can be characterized as intermediate or high and this appears to have remained unchanged through to 2010 (Table 1). We also looked at the potential effect of rainfall patterns on observed trends, an important secular driver of malaria transmission. However, at the hospital sites selected here, rainfall patterns did not have any apparent influence; the difference in rainfall remaining largely stable across the time series with the exception of 2005 when annual rainfall amounts were lower than customary across all the four sites.

Historically, Malawi has been a front runner in the fight against malaria, where following widely documented chloroquine resistance in Africa, Malawi was the first country to make the switch to SP and was also the first country to officially recommend SP for use in preventing malaria during pregnancy. However, between 2000 and 2006 there was a slow adoption of internationally agreed package malaria interventions of known high protective efficacy, ITNS, ACTs and IRS (Table 3), in part a direct result of inadequate overseas development assistance (ODA) during this period and a reliance on private-sector and clinic-based delivery of ITNs. Then funding landscape changed in 2006 and now Malawi has been granted slightly over $140 million in malaria ODA since 2002 and this has translated into an average annual external commitment to malaria funding of approximately $2.2 per person at risk in Malawi by 2010. National campaigns to deliver ITN free-of charge to at risk groups were started after 2007. However, mass ITN distribution campaigns have not raised coverage to effective levels anticipated to impact upon transmission [62]; reaching a coverage (in all ages) of between 24% and 33% in 2010, and still falls short of proposed targets (MSP 2005–2010). Additionally, effective implementation of the new AL drug policies only occurred in 2008 such that even though ITN and IRS coverage has improved with time, in 2010 the proportions of reported paediatric fevers in the last 14 days that were treated with an ACT at any stage of the illness remained suboptimal with more than 70% of children treated in a manner not recommended by national standard treatment guidelines.

Trends reported here are similar to reports from other high transmission sites in Uganda and Kenya [42], [68] and similar to previous reports from Malawi [33], further reinforcing the fact that trends in paediatric malaria hospitalization differ within a country and between countries across Africa. Importantly all is not equal and declining malaria burdens are not a ubiquitous consequence of RBM in Africa. It is likely that reductions in disease burden depend on some underlying features of malaria and levels and success of control coverage at individual sites. In this study series Salima and Mwanza are located in predominantly high transmission areas whereas Zomba and Rumphi are located in what would be defined as intermediate transmission zones [69]. Models of malaria intervention impact on future predicted levels of malaria endemcitiy suggest that bigger impacts are likely on PfPR_2–10_ when starting endemicity is lower following almost universal ITN coverage (circa 80%) within five years than when starting endemicity is higher [62]. Previous observations of hospital admissions in Western Kenya and Uganda [41], [42], [68] suggest that these areas of high transmission have not benefited from moderate scaling of ITN coverage as much as areas where historical transmission intensity is lower along the Kenyan coast [15], [16], [41]. Similarly, the data presented here from four Malawian hospitals also suggests that a combination of moderate-to-high transmission intensity and moderate coverage of ITN (24 to 33%) has not impacted on disease incidence.

One major caveat to our retrospective analysis of hospital-based data on clinically diagnosed paediatric malaria admissions is that we cannot be certain that each recorded case of “malaria” was in fact malaria. Parasitological confirmed cases of malaria are more common among patients admitted to hospital than those treated as out-patients, and therefore represent a more specific clinical temporal series but testing is never universal; laboratory records were incomplete and not linked to ward registers; and often incorrect where technologists err on the side of caution and record “scanty” for slides without parasites. This represents a perennial problem for all long-term hospital data on malaria in Africa and applies to all analyzes of hospital malaria trends reported to-date [4], [7], [8], [11], [14], [15], [17], [42], [70]–[75]. We believe however that these data do provide some information, albeit to be interpreted cautiously, about the temporal patterns of malaria burdens in the communities hospitals serve. First, despite a lack of sensitivity we compare what is clinically diagnosed as “malaria” among children admitted to a hospital with what admitting physicians regard as “non-malaria”. While in itself this does not legitimize the diagnosis of malaria it provides some internal, temporal check within hospitals. Second, we have examined the same features between hospitals adding external validity to the clinically diagnosed trends in “malaria”. Third, it is important to improve plausibility of observations by examining against other studies in Malawi, with better long-term diagnostic facilities, for example at the Queen Elizabeth Hospital in Blantyre [33], to which our findings correspond. And finally, it is important to triangulate against other data on malaria and its trends within the communities served by the hospitals. With respect to the later we feel that the observation that transmission intensity, as judged by mean parasite prevalence, has not changed during the period of our clinical “malaria” assemblies adds external plausibility to the observations of hospitalized “malaria”. What all studies on malaria hospitalization demonstrate is that there is an urgent need to improve upon the quality of parasitological diagnosis to improve the validity and utility of these data as measure of the changing burden of severe, potentially life-threatening disease across Africa.

Malawi has only enjoyed reasonable levels of ITN coverage for a few years and the impact on transmission and subsequently disease may take several more years to become evident. What is clear is that current coverage has not had a dramatic impact and that additional measures, as proposed in Malawi, like IRS combined with higher levels of ITN are necessary to meet the ambitions of the latest national malaria strategy to reduce by 50% the 2010 malaria burden. This will require an increase in malaria ODA and a more targeted plan of action and in order to document future epidemiological changes a result of increased ODA, better surveillance and better data.

## Supporting Information

Figure S1 **Map of clusters from the Malawian DHS dates of 2000, 2004 and 2010 within a 40 km radius of each hospital extracted in ARCGIS 9.3 (ESRI, Inc., Redland, CA, USA) to provide data with which to define ITN/IRS coverage within hospital catchments.**
(DOCX)

Table S1 **Temporally aggregated paediatric admission data for malaria admissions data and for all cause admissions in each of the 4 hospitals between 2000–2006 and 2007–2010 expressed per 1000 children aged 0–4 years per annum and 95% confidence intervals computed using a Poisson distribution.** Percentage change is the change in average annual malaria admission rates or average annual all-cause admission rates between the first (2000–2006) and the second (2007–2010) period.
(DOCX)
