# Supplementary material for: Childhood Malaria Admission Rates to Four Hospitals in Malawi between 2000 and 2010
Source: PLoS One. 2013 Apr 26;8(4):e62214. doi: 10.1371/journal.pone.0062214 (PMC3637378; doi:10.1371/journal.pone.0062214)

**Figure S1: Map of clusters from the Malawian DHS dates of 2000, 2004 and 2010 within a 40km radius of each hospital extracted in ARCGIS 9.3 (ESRI, Inc., Redland, CA, USA) to provide data with which to define ITN/ IRS coverage within hospital catchments.**


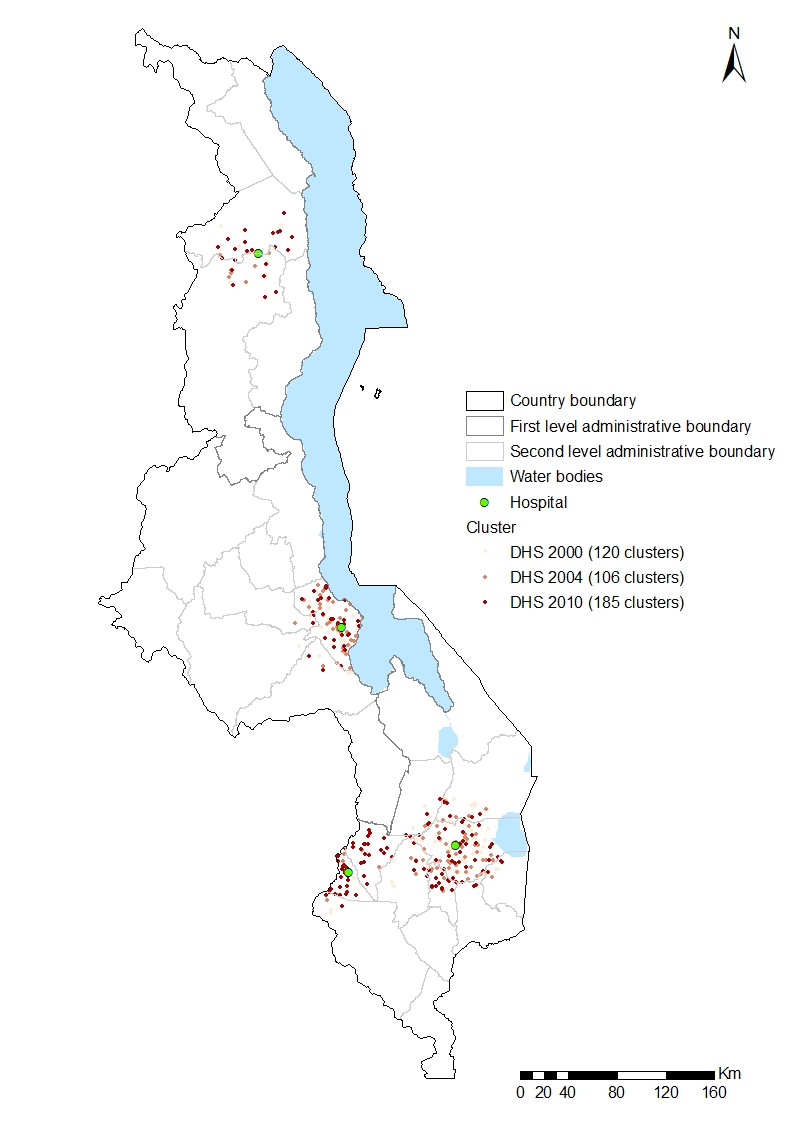

Supplement: Figure S1 — Map of clusters from the Malawian DHS dates of 2000, 2004 and 2010 within a 40 km radius of each hospital extracted in ARCGIS 9.3 (ESRI, Inc., Redland, CA, USA) to provide data with which to define ITN/IRS coverage within hospital catchments. (DOCX) [file pone.0062214.s001.docx]
