# Supplementary material for: Childhood Malaria Admission Rates to Four Hospitals in Malawi between 2000 and 2010
Source: PLoS One. 2013 Apr 26;8(4):e62214. doi: 10.1371/journal.pone.0062214 (PMC3637378; doi:10.1371/journal.pone.0062214)
Supplement: Table S1 — Temporally aggregated paediatric admission data for malaria admissions data and for all cause admissions in each of the 4 hospitals between 2000–2006 and 2007–2010 expressed per 1000 children aged 0–4 years per annum and 95% confidence intervals computed using a Poisson distribution. Percentage change is the change in average annual malaria admission rates or average annual all-cause admission rates between the first (2000–2006) and the second (2007–2010) period. (DOCX) [file pone.0062214.s002.docx]

**Supplementary Information**

**Table S1: Temporally aggregated paediatric admission data for malaria admissions data and for all cause admissions in each of the 4 hospitals between 2000-2006 and 2007-2010 expressed per 1000 children aged 0-4 years per annum and 95% confidence intervals computed using a Poisson distribution. Percentage change is the change in average annual malaria admission rates or average annual all-cause admission rates between the first (2000-2006) and the second (2007-2010) period.**

| **Hospital**  **[Map location]** | **Average annual all-cause admission rate 2000 -2006**  **[95% CI]** | **Average annual all-cause admission rate 2007-2010**  **[95% CI]** | **Change (%)** | **Average annual reduction in**  **All-cause admission rates per 1000 children 0-4 pa.**  **2000 -2010^1^** | **Average annual malaria admission rate**  **2000 -2006**  **[95% CI]** | **Average annual malaria admission rate**  **2007-2010**  **[95% CI]** | **Change**  **(%)** | **Average annual reduction in**  **malaria admission rates per 1000 children 0-4 pa**  **2000 -2010^1^** |
| --- | --- | --- | --- | --- | --- | --- | --- | --- |
| Rumphi [26] | 636.64 [612.26-661.76] | 444.62 [425.97-463.90] | **-30** | -23.69 | 285.36 [269.10-302.36] | 253.47 [239.59-267.97] | **-11** | -2.87 |
| Salima [27] | 212.67 [204.98-220.57] | 377.52 [367.94-387.29] | **+78** | 35.13* | 73.71 [69.29-78.34] | 203.90 [196.87-211.11] | **+177** | 22.59* |
| Mwanza [28] | 517.99 [498.22-538.34] | 652.38 [632.49-672.74] | **+26** | 20.37* | 288.93 [274.21-304.24] | 329.80 [315.69-344.38] | **+14** | 3.53 |
| Zomba [29] | 501.95 [493.08-510.94] | 646.44 [637.16-655.82] | **+29** | 13.37 | 153.14 [148.26-158.14] | 204.28 [199.09-209.58] | **+33** | 4.24 |

Notes

^1^ Summary of trend per year for each admission series over the 11-year period 2000-2010: Showing the coefficient of *trend*, in each site, represents the annual trend i.e. the change in admission rates per 1000 children aged 0-4 years per annum. Admissions rates declined (-) or increased (+). The asterix denotes the statistical significance of the trend.

* Significant P-value <0.05

** Borderline significant P-value between 0.05 to 0.07
